# Dairy cows’ motivation to nurse their calves

**DOI:** 10.1038/s41598-024-64038-z

**Published:** 2024-06-14

**Authors:** Emma Hvidtfeldt Jensen, Melissa Bateson, Heather W. Neave, Jean-Loup Rault, Margit Bak Jensen

**Affiliations:** 1 Department of Animal and Veterinary Sciences, Aarhus University, Tjele, Denmark; 2 Biosciences Institute, Newcastle University, Newcastle upon Tyne, UK; 3 Institute of Animal Welfare Science, University of Veterinary Medicine Vienna, Vienna, Austria; 4 Present Address: Department of Animal Sciences, Purdue University, West Lafayette, Indiana USA

**Keywords:** Animal behaviour, Behavioural ecology

## Abstract

When weaning offspring, female mammals limit nursing opportunities. This study aimed to investigate whether imposing a gradual reduction in daily contact time, by separating cows from their calves as an attempt to stimulate weaning, reduced dairy cows’ motivation to nurse their calves. For seven weeks, 84 Holstein–Friesian cow-calf pairs were housed with either full-time (23 h contact/d), part-time (10 h contact/d), or no contact. In the following two weeks, half of full- and part-time pairs were subjected to reduced contact (50% of initial contact in week 8, 25% of initial contact in week 9), while the other half continued with unchanged contact. In weeks 8 and 9, cows’ motivation to obtain full contact to and opportunity to nurse their calves was measured using weighted push gates using a novel maximum price paid method providing an alternative choice to the cows to reduce frustration. Cows with reduced calf contact were more motivated than cows with unchanged contact; however, cows used the alternative choice less than expected. The results show that cows’ motivation for full calf contact and opportunity to nurse increases when daily calf contact is reduced, illustrating that dairy cows are motivated to continue nursing their 9- to 10-week-old calves.

## Introduction

In a natural setting, cows gradually wean their calves after several months of nursing^1,2^. However, in conventional dairy production systems, calves are commonly separated from their dams within hours or days of calving and artificially fed milk until 7 to 12 weeks of age (e.g., refs.^3–5^). There is an increasing interest in the animal welfare implications of prolonging cow-calf contact (CCC) from both consumers, farmers, and researchers^6–11^. Even with prolonged CCC, weaning of calves is still targeted at two to five months after calving^11^, which is earlier than in a natural setting. Most research on CCC and stress at separation regards the impact on calves (reviewed by refs.^9,10^), however, evidence also suggests that cows’ welfare is affected (e.g., refs.^12,13^).

Using a so-called stepwise weaning strategy, where the termination of nursing and the loss of contact are separated in time, can reduce both cows’ and calves’ negative reaction to weaning^12,14,15^. For calves, the loss of milk is less stressful when they can still have physical contact with the cow, indicating that both milk and maternal contact are important aspects of their relationship to their dam^16^. For cows, the causation for reduced reaction is less clear, but the stress-reducing effect of stepwise weaning indicate that both social contact to the calf and nursing are important aspects of the maternal motivation. The cow is expected to maintain her motivation to nurse her calf, until the calf becomes nutritionally independent and can sustain itself on solid feed^17^

Part-time CCC^18^ reduces nursing duration^19^; however, calf growth was similar between full- and part-time systems^14^, possibly indicating similar milk intake. Nevertheless, calves with part-time cow contact spent more time eating solid feed than calves with full-time contact^19^, which could possibly reflect that calves compensate for reduced milk intake and may indicate that part-time calves obtain nutritional independence sooner than full-time calves. In a natural setting, the female mammal gradually weans her offspring by reducing her parental investment, e.g., by reducing milk availability^20,21^. Among cattle living in semi-natural environments, cows’ time spent nursing gradually decreases as the calf ages^22,23^ until the calf is weaned off milk at six to 11 months^1,2^. Conventionally reared calves can be encouraged to increase their solid feed intake by reducing their daily milk ration^24^. In a CCC system, gradually reducing daily cow-calf contact, and thus calves’ access to suckle, may also encourage the calf’s nutritional independence. This effect may be further enhanced in a part-time CCC system. Whether part-time CCC and gradually reducing cow-calf contact also reduce the cow’s motivation to nurse is yet unclear. To our knowledge, no studies have investigated the cows’ motivation to nurse nor the effect of daily contact duration, or gradually reducing daily contact duration, on nursing motivation. This study therefore aimed to investigate the importance of nursing to dairy cows with either full- or part-time calf contact, and how this is affected by gradually reducing calf contact and thus nursing opportunity. We hypothesized that cows’ motivation to nurse their calves would decrease with increased nutritional independence in the calves, i.e., that cows with part-time calf contact would be less motivated to nurse their calves than cows with full-time contact at eight to nine weeks postpartum, and that cows with no calf contact would be the least motivated to nurse. Furthermore, we hypothesized that nursing motivation would be reduced by gradually reducing the CCC during the final two weeks before complete separation.

Different methods exist to assess animal motivations. Some methods require consecutive testing within a session and thus reward periods that can be divided into smaller bouts^25^. However, some rewards lose value by being divided, and in such cases, consecutive testing is not feasible. We expected nursing behavior to be devalued by interruption. A recommended method for undividable behaviors, or resources, is the maximum price paid (MPP) method^26,27^. However, the MPP method may induce frustration, as it limits the animal’s ability to control its environment^28–30^; a secondary aim of this study was therefore to evaluate a novel take on the method. In this novel approach, the cows had the choice between full contact with their calf, where nursing was possible, and partial contact, where most maternal behaviors, except for nursing, were possible. Previous studies have found that partial contact is less attractive than full contact (e.g., calves^31^ and horses^32^). However, we expected partial contact to be able to partly substitute^33^ for full access to the calf. In the current study, the work required to access partial contact remained low, and partial contact was thus meant to function as a cheaper, albeit less attractive, substitute to full contact. Providing the cows with a choice gives them more control over the test situation, which is expected to mitigate frustration^34^. We therefore hypothesized that cows would utilize the option for partial contact rather than wait for the test to be over, when the work required to access full contact exceeded their maximum price.

## Results

### Training

Of the 81 cows enrolled for training, 14 of 14 full-time reduced contact (RC), 13 of 14 full-time unchanged contact (UC), 15 of 15 part-time reduced contact, 12 of 12 part-time unchanged contact, and 13 of 26 no-contact cows met the learning criteria. Fewer no-contact cows than any of the other treatments passed training (full-time UC, *P* = 0.01; full-time RC, *P* = 0.001; part-time UC, *P* = 0.003; part-time RC, *P* = 0.001). No difference in training success was found between any of the other treatments.

### Maximum price paid

**Figure 1:**
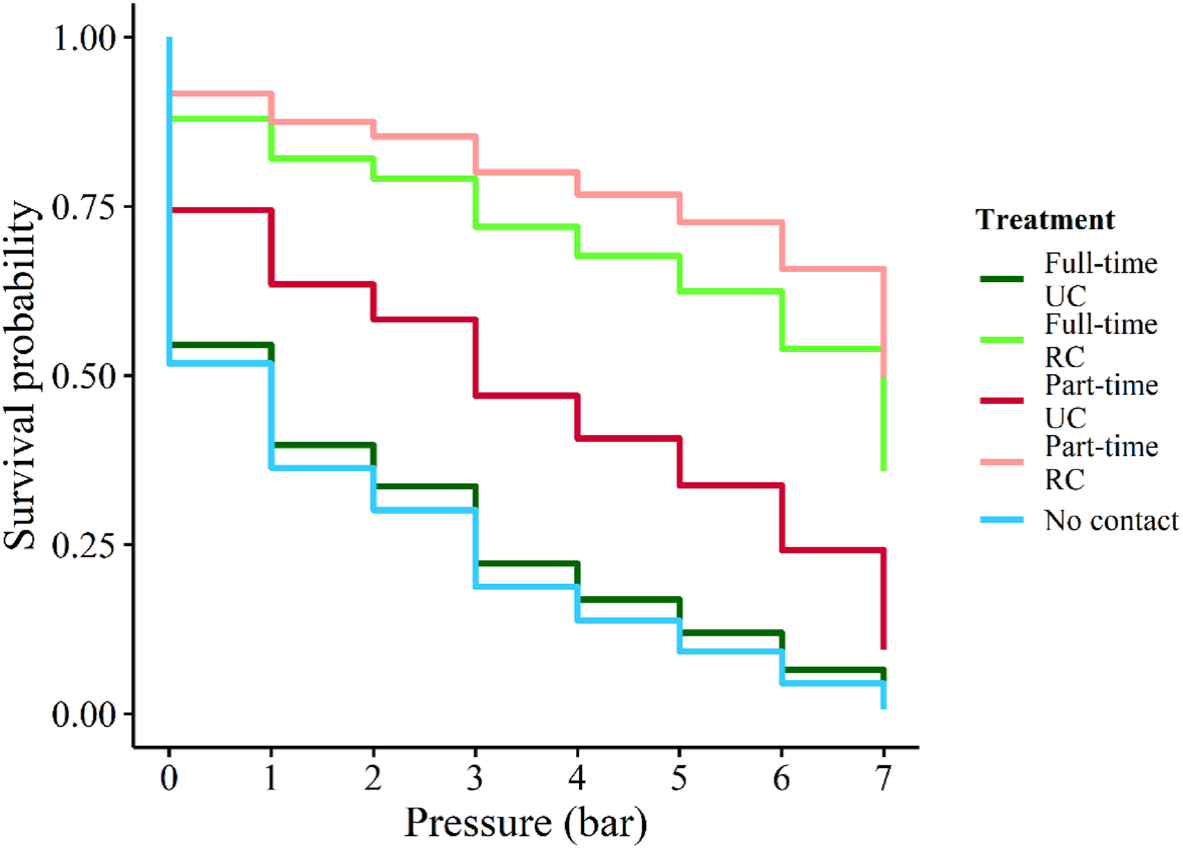
Survival curves for the five treatments. Sixty-eight Danish Holstein–Friesian cows were tested approx. 8.5 weeks postpartum. No-contact and full-time cows with unchanged contact (UC) had significantly lower survival probability as pressure increased than both full- and part-time cows with reduced contact (RC) and therefore a lower maximum price paid. Part-time cows with UC did not significantly differ from any of the other treatments. Cows pushing through 8.0 bar could in theory have a higher maximum price than what we were able to detect in our set-up. Therefore, survival probability at 8.0 bar was not computable, and the x-axis does not go further than 7.0 bar. The survival probabilities of both RC treatments and part-time UC did not reach 0 at 7.0 bar of pressure, indicating that some cows on these treatments were likely to have even higher MPPs than what we could measure.

The mean maximum price paid (MPP) was 5.29 ± 3.4 bar for full-time reduced contact cows, 1.85 ± 2.2 bar for full-time unchanged contact cows, 6.2 ± 2.8 bar for part-time reduced contact cows, 3.17 ± 3.1 for part-time unchanged contact cows, and 1.62 ± 1.8 for no-contact cows. For corresponding weights, see Table 1. Fourteen of 68 cows (5 full-time RC, 7 part-time RC, and 2 part-time UC) passed through the maximum weight of 8.0 bar. Maximum price paid was affected by treatment (Cox’s proportional hazards mixed effects model: Χ^2^ = 25.9, *df* = 4, *P* < 0.001, Fig. 1). No-contact cows had a larger hazard rate, and thus a higher probability of reaching their MPP earlier, than full-time reduced contact (ratio and CI 2.78 [1.51–5.13] vs. 0.43 [0.22–0.84], z-ratio = 3.69, *P* = 0.002) and part-time reduced contact (0.34 [0.18–0.65], z-ratio = − 4.01, *P* < 0.001). Similarly, full-time unchanged contact cows also had a higher hazard rate (2.56 [1.39–4.69]) than both full-time reduced contact (z-ratio = 3.43, *P* = 0.006) and part-time reduced contact (z-ratio = − 3.92, *P* < 0.001), meaning that full-time unchanged contact cows had lower MPPs than full- and part-time reduced contact cows. No-contact and full-time unchanged contact cows were not different from each other. Likewise, full- and part-time reduced contact cows were not different from each other. The hazard rate, and thus the MPP, of part-time unchanged contact cows (1.22 [0.67–2.23]) did not differ from any of the other treatments.

**Table 1:** The level of resistance that cows would experience at each pressure level before the pressure on the gate would release.

Pressure (bar)	Weight (kg ± std)
1	29.9 ± 0.9
2	42.7 ± 0.9
3	55.8 ± 0.7
4	68.9 ± 0.6
5	82.1 ± 0.8
6	95.9 ± 1.1
7	111.2 ± 0.4
8	124.6 ± 1.2

The procedure for recording corresponding weights is described in Supplementary Material 1.

Full contact was chosen in 253 of 360 total test sessions (70.2%), partial contact in 65 sessions (18.1%), and cows remained in the start box in 42 test sessions. Latency to pass the gate was recorded for all instances of cows choosing full contact; however, five latency records were omitted from the analysis due to mistakes in the time recordings, resulting in data from 248 test sessions. Descriptively, part-time reduced contact cows had the lowest latency to choose full contact (average ± standard deviation; 4.6 ± 6.9 s) followed by full-time unchanged (5.0 ± 3.4 s) and reduced contact (5.0 ± 7.6 s), no-contact (17 ± 24 s), and part-time unchanged contact cows (23.3 ± 26.7 s). To evaluate whether the two minutes were a long enough period for cows to make a choice, we investigated in how many test sessions latency to reach full contact was under 60 s, and found this to be the case in 229 (92.3%) of test sessions.

### Nursing

Every time a reduced contact cow chose full contact, nursing took place (74/74 sessions for full-time cows, 93/93 sessions for part-time). This was more than what was observed for the other treatments (4/24 sessions for full-time UC, 2/38 sessions for part-time UC, and 6/21 sessions for no-contact cows; chi squared test: Χ^2^ = 203.2, *df* = 4, *P* < 0.001).

### Substitutability between full and partial contact

Twenty-three cows had two consecutive fails. Due to this low number of animals, we analyzed the level of substitutability across all treatments instead of between them. To investigate whether cows preferred partial contact over remaining in the start box, we defined the null hypothesis that cows were equally likely to choose these options. For the cows’ first fail, the null hypothesis could not be rejected. Nevertheless, for the second fail, cows were less likely to choose partial contact than to remain in the start box (binomial test: probability and CI 0.26 (0.10–0.48), *P* = 0.035). Additionally, we did not detect any relationships between the cows’ choice (partial contact or to remain in the start box) and whether they first attempted to reach full contact (Table 2). Finally, we evaluated the consistency of cows’ choice between the first and second fail. Of the 23 cows with two consecutive fails, 6 always chose partial contact, 14 always remained in the start box, and 3 cows switched between the two options. All three switching cows initially chose partial contact but stayed in the start box for their second fail.

**Table 2:** Use of alternative options in the cows’ first and second failed attempts to reach full contact.

Alternative choice	Tried full contact	Did not try full contact	Total
First fail			
Chose partial contact	2	7	9
Stayed in start box	5	9	14
Total	7	16	23
Second fail			
Chose partial contact	1	5	6
Stayed in start box	1	16	17
Total	2	21	23

Only cows that passed 1.0 bar of pressure and who had two consecutive failed attempts are included. Also shown is whether the cows first tried to access full contact (i.e., touched the push gate with their shoulders) before they chose either partial or no contact, i.e., stayed in the start box.

## Discussion

Contrary to our hypothesis, reducing calf contact first to 50% and then to 25% of the original contact time did not simultaneously reduce cows’ motivation to nurse at eight to ten weeks postpartum. Instead, full-time cows with unchanged contact (UC), who had 23 h/d of calf contact, showed similar motivation as cows who had been separated from their calves for more than eight weeks. No-contact cows’ low motivation to obtain full contact is supported by their lower rate of meeting the learning criteria during training. Trainability can be used as a proxy for motivation, as highly valued rewards are expected to increase training success^35^. Reducing daily contact significantly increased full-time cows’ motivation to nurse, while the observed increase was not significant for part-time cows, who initially had 10 h/d of calf contact. Full- and part-time reduced contact (RC) cows continued to push through the gates to access and nurse their calf for longer, and thus reached a higher maximum price, than no-contact cows and full-time UC cows; part-time UC cows were intermediate. By reducing the time available for maternal-filial contact, it appeared that the opportunity to nurse became even more valuable for full-time cows during this period, as the cows’ access to their calf and their opportunity to nurse became more restricted. Nevertheless, in a concurrent study including the same experimental animals as the present one, reducing contact time first to 50% and then to 25% did not consistently reduce the time that cows spent nursing in the home pen^36^. The more intense nursing during the time that cow and calf were together supports the high motivation to nurse that was measured in the present study.

The initial contact time did not affect nursing motivation, as no difference was found when comparing full- and part-time treatments within the cows experiencing either reduced or unchanged contact. Apparently, part-time contact did not in itself reduce nursing motivation which probably did not better prepare cows and calves for weaning off milk; this is supported by reports of similar reactions to weaning in cow-calf pairs housed with full- and part-time contact^13,14^. On the other hand, reduced contact appeared to increase nursing motivation (significantly for full-time and numerically for part-time). This increase may be an effect of time since last nursing bout, as cows were tested in the morning before reduced contact pairs were reunited. Nursing had therefore been prevented for at least 11 h for any of the reduced contact pairs before testing, while unchanged contact pairs had only been separated for 1 h. That part-time reduced and unchanged contact cows only differed numerically from each other could be caused by part-time unchanged contact cows already experiencing restricted calf access, while full-time reduced contact cows experienced a substantial reduction in calf contact compared to the nearly unlimited calf access of full-time unchanged contact cows.

Prolonging the period of reduced contact or eliminating nursing, e.g., by fence line weaning^15^, might have reduced nursing motivation. To our knowledge, we are among the first to investigate a weaning method aiming to gradually decrease nursing^18^; it is therefore difficult to determine the ideal length of the reduced contact period. It is expected that natural weaning, i.e., when the cow begins to reject suckling attempts, starts when milk intake meets less than 40 to 50% of the calf’s nutritional requirements^16^. In future studies, recordings of the solid feed intake of calves with reduced contact could provide an indication of when separation could take place. Studies on beef cattle have shown that prevention of nursing does not immediately break the mother-filial bond; however, it does affect both cows and calves’ motivation to stay in close proximity to each other after a few days of nursing prevention^15,37^. Additionally, Salers cows were only rejecting nursing attempts after 20 days of separation^38^, suggesting that to reduce nursing motivation and weaken the mother-filial bond, nursing must thus be prevented for at least 20 days. This was not possible in the current set-up. Increasing the period of nursing prevention would likely mean that the calves would be older at separation; this may also contribute to a reduced reaction at separation. Delaying weaning in artificially reared dairy calves results in better growth and reduced stress reaction^39^.

**Figure 2:**
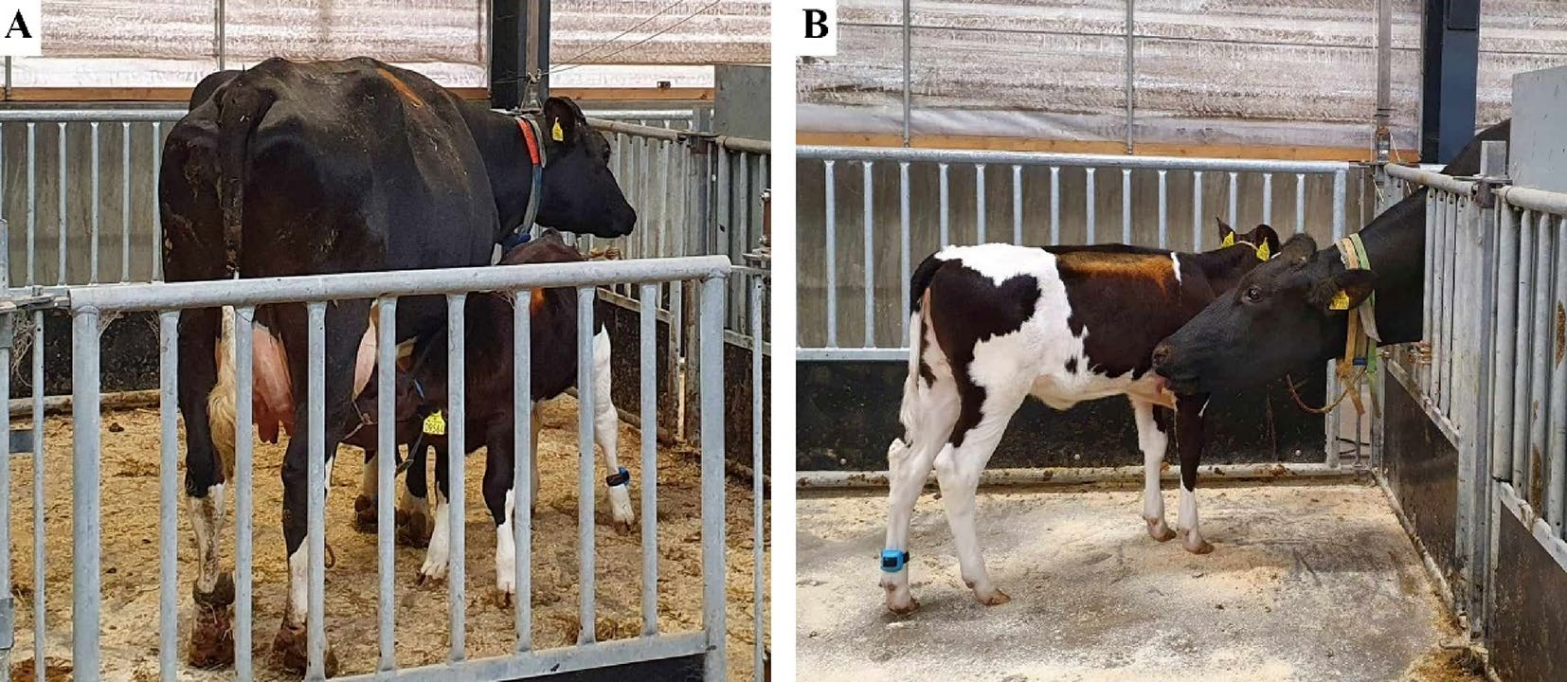
The two options that the cow could choose between in the test situation. (**A**) full contact; cow and calf are together in the same reward pen, nursing is possible. (**B**) partial contact; cow and calf are in two different reward pens, but the cow could reach the calf through the “window”; maternal behaviors such as sniffing and licking the calf were possible, but nursing was not. Photo credits: EHJ.

In the current test set-up, cows could choose between full calf contact, where nursing was possible, partial calf contact, where nursing was not possible, or no calf contact (staying in the start box). In this design, we assumed that motivation to obtain full calf contact is similar to the motivation to nurse, but this may not be the case. In the training process, we noted that all cows entering the reward pen with partial contact found the “window” that allowed them to reach their calf. We therefore assume that the cows learned that they could touch, sniff, and lick the calf (Fig. 2), even if they did not obtain full contact. Cows were thus able to perform a range of maternal behaviors through the window, except nursing. Other studies have shown that animals are able to differentiate between full and partial contact (e.g., calves^31^ and horses^32^). We therefore assumed that the cows also were able to make this distinction. However, cows did not utilize the partial contact option as much as we had expected. Perhaps they had not understood that both gates were available to them. However, during training, cows were deterred from passing gates by a wooden board, which both visibly and physically blocked the gate, while both gates were “open” both physically and visibly during testing. Furthermore, cows had all participated in a similar experiment shortly before the current study^40^ and had in that connection experienced multiple free choices. For their first failed attempt to reach full contact, we did not see a clear preference between partial and no contact, i.e., remaining in the start box. For the second failed attempt to reach full contact, cows were more likely to remain in the start box than to choose partial contact. All cows who changed their response between first and second fail switched from partial calf contact to no calf contact, showing that even though they had understood that the alternative gate was available, they preferred to remain in the start box. These results indicate that partial contact does not substitute for full contact^33^ and that nursing motivation is an important aspect of maternal motivation. Although we cannot rule out that full contact allows for better opportunity for other maternal behaviors than nursing, which may explain why cows continuously chose this option even when calves did not suckle, the high occurrence of nursing supports that nursing opportunity is an important aspect of full contact.

Previously, cows have been observed to vocalize when prevented from nursing their calves^12,15,37^. In the current study, cows were anecdotally seen assuming a “nursing position”, a characteristic stance with spread hind legs, possibly in attempt to encourage suckling (also observed by ref.^12^). Calves did not always respond to this by suckling, and whether suckling encouragement is the function of this position should be investigated in future studies. Whether nursing took place or not depended not only on the cow, but also on the calf and the calf’s hunger level or experience with suckling. With reduced suckling opportunity on the reduced contact treatments, we expected the calves to compensate for reduced milk intake by eating more solid feed^24^. This could make the calves less reliant on milk from their dams, and thus reduce their motivation to suckle. However, reduced contact did not consistently reduce suckling time, indicating that calves were still nutritionally dependent on their dams^36^. Furthermore, suckling the dam results in high oxytocin levels^41^, likely motivating the calves to suckle as much as possible, even if alternative food sources are available. This may explain why suckling was more likely to take place for the cow-calf pairs with reduced contact.

Reduction of suckling may lead to accumulation of milk in the udder. This increases the intramammary pressure, which causes discomfort for the cow^42^. To reduce discomfort, cows may be motivated to seek out opportunities for their calves to empty their udder through suckling. However, the cows in the current study were still nursing their calves and were also machine-milked twice daily. It is therefore unlikely that they would have experienced high intramammary pressures, as the udder takes 25 to 30 h to fill to full capacity^43^. Furthermore, a previous study found that cows show low motivation to be milked, and only start visiting an AMS, when they receive a food reward^44^, further indicating that aspects other than just emptying the udder must affect the motivation to nurse. Cows nursing calves have higher levels of oxytocin than cows being machine milked^41,45^. Increased levels of oxytocin are often linked to positive or stress-coping situations (reviewed by refs.^46,47^) and social behaviors (reviewed by ref.^48^). This could indicate that nursing is a positive experience to the cows; however, the relationship between oxytocin and positive affective states is still somewhat unclear^48^. That the cows in the current study were motivated to obtain full contact, and thus nursing opportunity, could further support that nursing is a pleasant experience to the cow. We encourage future research to utilize additional indicators of positive affective states to further investigate this.

Overall, our results indicate that our gradual weaning and separation method was not successful in reducing cows’ motivation to nurse and thus induce them to wean their calves off milk. Commercially managed dairy cows have ad libitum access to food, water, and shelter, they experience low predation risk, and they efficiently produce large amounts of milk. Therefore, the cow's incentive to wean her calf is probably low, at least when the calf is at the young age of 8.5 weeks, as in the current study. Weaning at this age is much earlier than the expected natural weaning age (6 to 11 months^1,2^), and calves could be expected to still rely mostly on their mothers’ milk for nutrition. There is thus little basis for a parent–offspring conflict at this time after calving^21^.

The current study is subject to some limitations. Ideally, the cognitive and physical abilities of the cows should have been assessed and balanced across treatments. Some cows may have had difficulties understanding the test set-up, as they may not have understood the choice between full and partial contact. Nevertheless, by implementing the learning criteria, only cows that had understood how to pass the push gates were included in the MPP test, which did control for some learning variability. Regarding the physical abilities of the cows, a better measurement may have been the weight pushed relative to the cow’s own weight. However, an MPP test does not aim to measure the maximum price the animal is able to pay, but rather the maximum price it is willing to pay; the weight on the gate should therefore never surpass the cows’ physical abilities. In a previous study using similar push gates, cows were able to pass gates of 258 kg-f^30^, which was much higher than the maximum weight available in the current study (Table 1). We do therefore not expect the maximum resistance cows experienced in the current study to surpass their physical abilities. Furthermore, as the distribution of parity across treatments was similar, cow size and experience were also assumed to be similar between treatments. As the method used in the current study is a novel take on the MPP method, a validation of the method is required, which our set-up did not allow for. Ideally, a parallel “conventional” MPP test could have been carried out, allowing the results of both methods could be compared. However, pilot studies revealed that cows showed reduced interest in their calf when their motivation was tested multiple times a day, preventing such a validation strategy. The effect of multiple tests a day also contributed to us running the motivation test across both steps of contact reduction, instead of doing separate tests in both week 8 and 9. Had we divided the test in two, we may had been able to detect an effect of 50% contact vs. 25%, however, having only a week to test the cows would force us to either reduce the maximum price available or to increase the price more drastically from day to day. Both strategies would reduce the sensitivity of the test, and thus possibly our ability to detect differences between the treatments. Finally, an important aspect we were not able to include was the behavior of the calf. A restless and vocalizing calf may have increased the cow’s motivation to obtain full contact. Nevertheless, as both cows and calves had participated in a similar study shortly before the current^40^, the calves were experienced with being tethered in the arena, and they were generally calm and quiet during testing. We do therefore not expect that calf behavior had a substantial effect on the current study.

In conclusion, cows with reduced calf-contact paid higher maximum prices than cows with unchanged calf contact. Additionally, cows reaching their maximum price utilized the option for partial calf contact less than what was expected by chance. This indicates that partial contact could not substitute for the properties of full contact and thus nursing opportunity. From our results, it appears unlikely that limiting calf contact induced weaning; rather it may have made calf contact and nursing even more valuable to the cows by limiting their access to these resources. On the other hand, motivation to nurse may have rather depended on time since last nursing bout; unfortunately, which explanation is most likely cannot be determined from the current study and deserves more research. Outside of the test, cows were still able to compensate for the reduced contact by nursing their calves more intensively. Perhaps if nursing had been completely prevented, nursing motivation would have decreased over the subsequent weeks. Nursing motivation is likely an important aspect of maternal motivation, since cows showed clear motivation to obtain full calf contact and nurse their calves. Future research should investigate how nursing motivation is affected by calf age, and if even longer periods of reduced contact could reduce nursing motivation.

## Materials and methods

This study was conducted from September 2021 to August 2022 at the Danish Cattle Research Center, Aarhus University (Tjele, Denmark). The use of the experimental animals was permitted by the owner of the animals, Aarhus University. All animal procedures were approved ethically by the Danish Animal Experiments Inspectorate in accordance with the Danish Ministry of Environment and Food Act No. 474 (May 15, 2014). The study was furthermore carried out in compliance with the ARRIVE guidelines.

### Housing and contact treatments

Eighty-four Danish Holstein–Friesian cow-calf pairs were enrolled in the study. Pairs were enrolled over seven blocks with 12 pairs in each block. Calving took place in individual calving pens, where cow and calf remained for approximately 48 h post-partum (range 42–66 h). To be enrolled, the calving had to be of a single, healthy calf, without assistance, and the calf had to suckle unassisted within 48 h. Pairs were allocated to one of three contact treatments based on their calving order; full-time calf contact (23 h/d, separated when cows were milked in the parlor), part-time calf contact (10 h/d, separated between afternoon and morning milking), or no contact (0 h/d, separated 48 h after calving). The no-contact treatment functioned as a control to the treatments with cow-calf contact. Two pairs at a time were allocated to each treatment, and the order of allocation rotated across the blocks. Enrollment took place over two weeks before the 10-week experimental period began.

**Figure 3:**
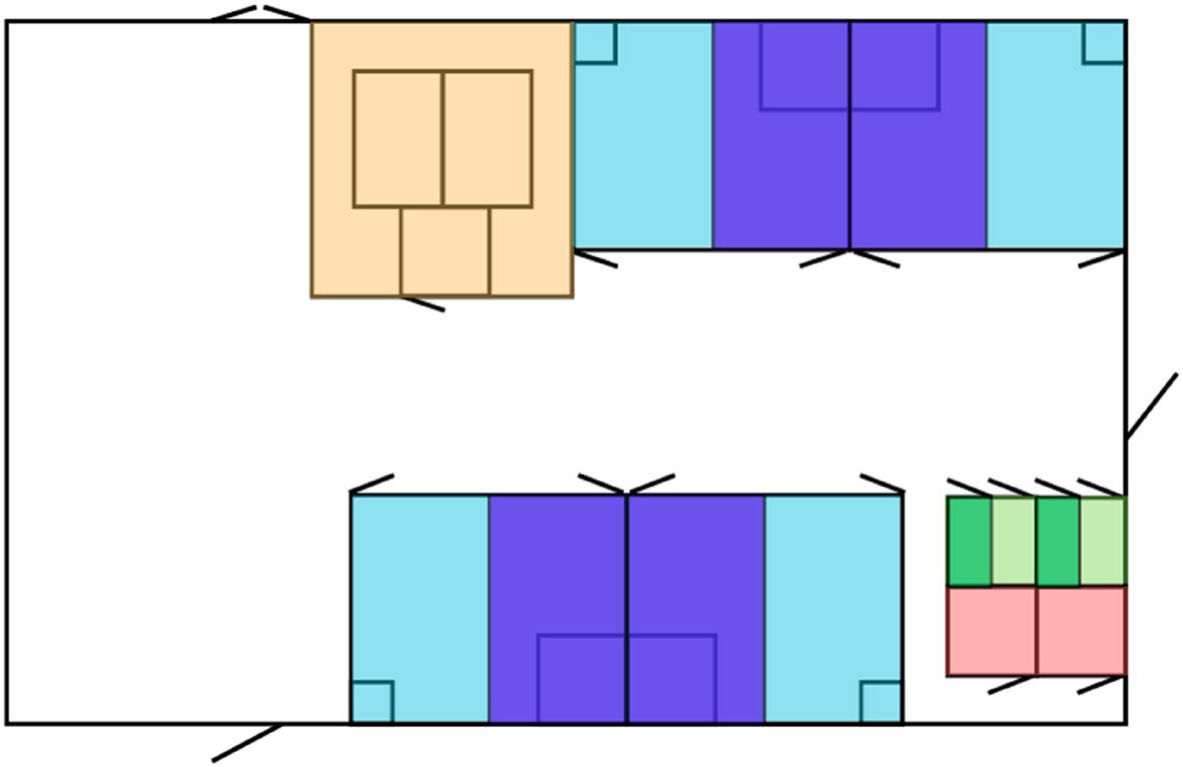
Schematic drawing of the barn set-up during the reduced cow-calf contact period. Contact pairs were housed in groups of two pairs in deep-bedded pens (7.5 × 4.5 m). Pairs with unchanged contact had access to the smaller calf creep (light blue), while pairs with reduced contact had access to the larger calf creep, where the calves were kept during the periods with restricted contact (dark blue). No-contact calves were housed in pens of two calves depending on whether they received milk ad libitum (light green) or a restricted milk ration (dark green); no-contact cows were housed in a separate barn (not pictured). For an hour before training and testing, contact calves were held in holding pens (red), and all cows were trained and tested in the same arena (orange).

During the main rearing period (week 1 to 7 of the experimental period), the two contact treatments (full- and part-time) were housed in separate straw-bedded pens (7.5 × 9.0 m), each housing four pairs. Each pen was fitted with two calf creeps (3.0 × 3.0 m and 1.5 × 1.5 m, Fig. 3). The pen sides dividing neighboring straw-bedded pens were solid, while pen front and sides of the calf creeps were made from tubular metal bars. No-contact cows were housed in a separate barn in a free-stall pen housing up to 12 cows at a time (8 experimental, 4 non-experimental cows). Between afternoon- and morning milking (approximately 15:30:00 to 05:30:00), part-time cows were housed in a free-stall pen adjacent to the no-contact cows. Part-time calves remained in the straw-bedded pen, thus preventing visual, olfactory, and auditory contact between the dams and calves. No-contact calves were housed in groups of four in straw-bedded pens (3.0 × 3.0 m) with sides made of metal tubular bars in the same barn as the contact treatments (Fig. 3). The no-contact calves received milk ad libitum in two daily feedings via teat buckets.

**Figure 4:**
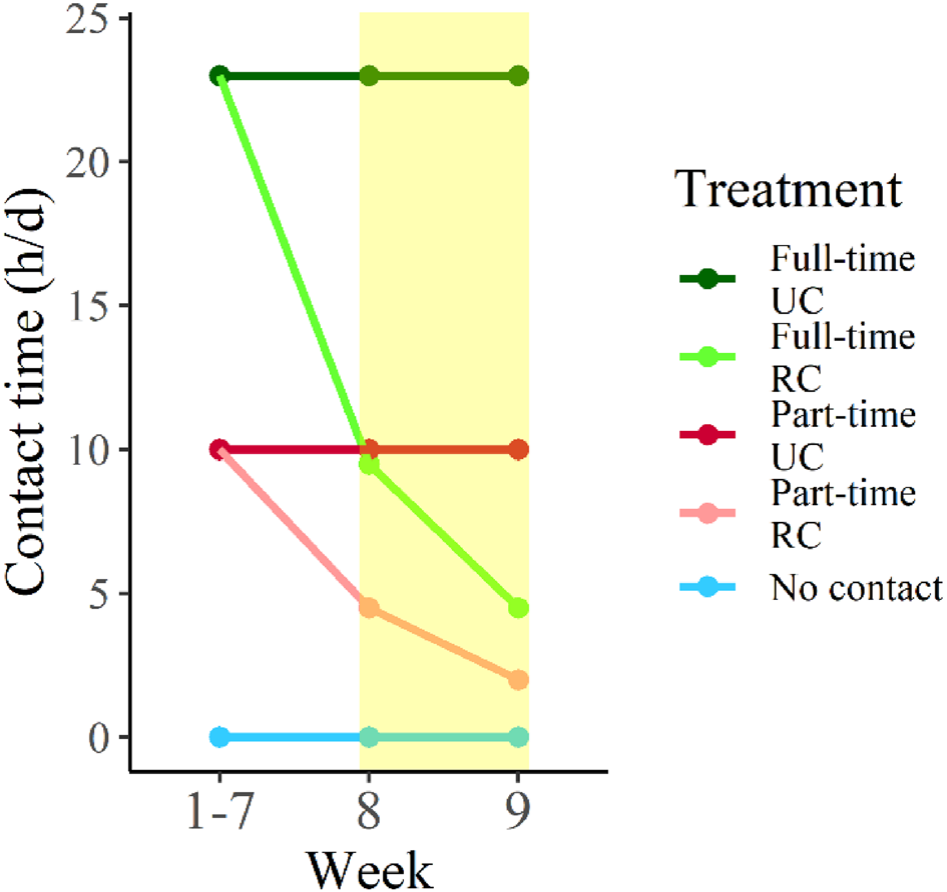
Amount of daily cow-calf contact for the different treatments across the experimental period. Nursing motivation was tested in week 8 and 9 (the yellow area). UC refers to unchanged contact, while RC refers to reduced contact.

In week 8 and 9 (the reduced cow-calf contact period), an additional contact treatment was added. Half of all contact pairs experienced a gradual reduction in contact time (RC), while the other half continued with unchanged contact (UC). The straw-bedded pens were split into two (7.5 × 4.5 m) using pen sides of tubular metal bars, with one half containing the small calf creep and the other containing the large calf creep (Fig. 3). Two pairs were randomly allocated to each of the new pens. Reduced contact was established by confining the calves in the large calf creep for approximately 50% (week 8) and 75% (week 9) of the original contact time (Fig. 4). Throughout the reduced contact period, all reduced contact calves were released from the calf creep at 11:00 h. In week 8, full-time reduced contact calves were confined in the calf creep again at 21:00 h, and part-time reduced contact calves at 15:30 h, when their dams went to the milking parlor. In week 9, full-time reduced contact calves were confined at 15:30 h, and part-time reduced contact calves at 13:00 h. While confined in the creep, calves had visual, olfactory, and limited physical contact with their dams; however, nursing was not possible. The unchanged contact treatments functioned as a control for the reduced contact treatments. Simultaneously, the no-contact calf pens were split in two, and half of the no-contact calves experienced a reduction in their daily milk allowance corresponding to 50% (week 8) and 75% (week 9) of their ad libitum milk intake. The other half continued with milk ad libitum in two daily feedings. As this study focuses on the cows’ perspective, and as the reduction in milk allowance did not directly affect the no-contact cows’ relationship with their calf, we will regard all no-contact pairs as one treatment. In week 10, contact cows and calves were fully separated from each other, and no-contact calves were all completely weaned off milk.

Sample size was based on availability of cow-calf pairs and was supported by a post hoc power calculation to detect significant differences in maximum price paid (5% significance level, power level > 90%). The distribution of cow parity was not fully counter-balanced but was similar between treatments (see Table 3).

**Table 3:** Number of cow-calf pairs, and cow parity and calf sex distribution across treatments.

Treatment	Parity		Calf sex		Pairs per treatment
	Primiparous	Multiparous	Heifers	Bulls	
Full-time UC	5	9	8	6	14
Full-time RC	6	8	9	5	14
Part-time UC	6	6	6	6	12
Part-time RC	6	9	7	8	15
No contact	10	16	14	12	26

Eighty-one cows were enrolled for the study. UC refers to unchanged contact, while RC refers to reduced contact.

For this study, the cows’ motivation to nurse their calf was investigated. Before the current study, cows and calves participated in other studies as part of a larger project. This included another motivational test, which took place in week 5 to 7 of the experimental period and utilized the same testing apparatus as the current study^40^.

### Test apparatus

**Figure 5:**
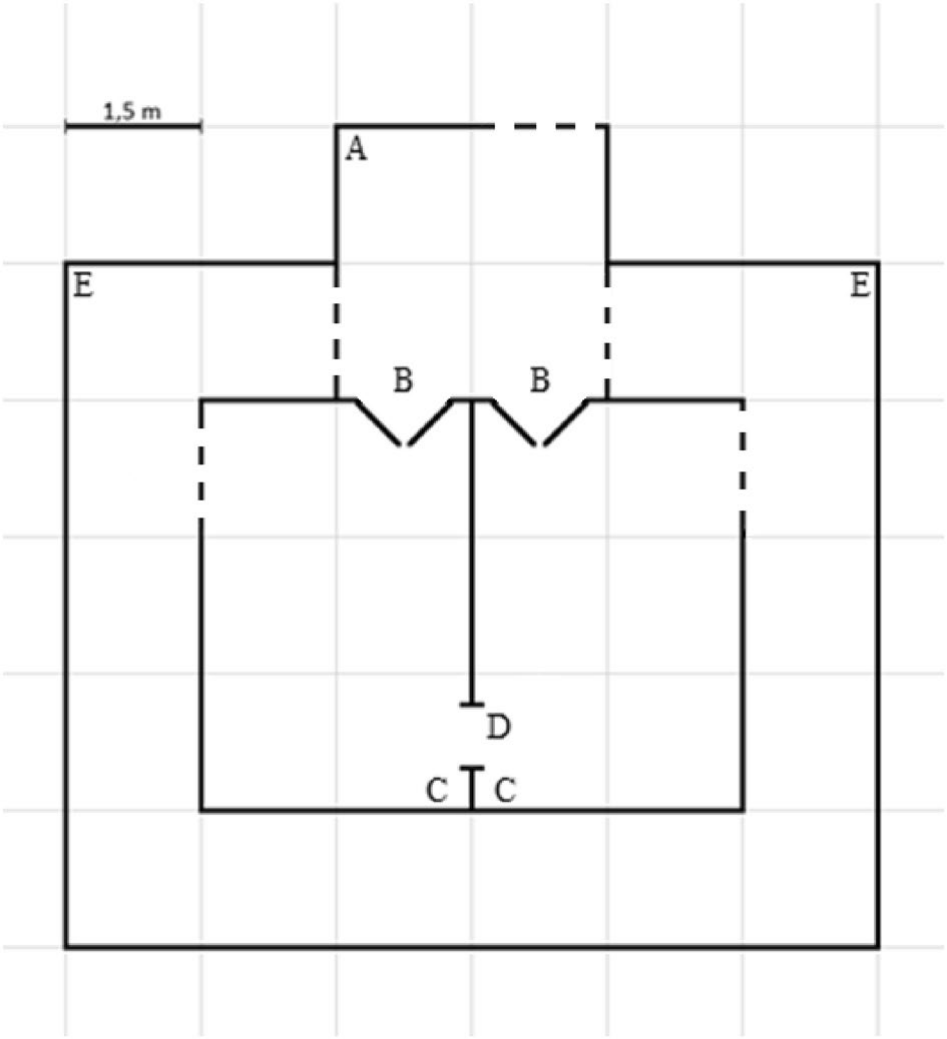
Schematic drawing of the test arena. (**A**) The start box. From here, the cow must choose between the two reward pens. (**B**) Push gates. The cow can see through each push gate. (**C**) Tether points inside reward pens. The calf is tethered either in the left or right pen, depending on what the cow has been trained to expect. (**D**) The “window”. This window allows the cow to sniff and lick the calf, even if she does not choose the reward pen wherein the calf is tethered. (**E**) Return walkways. Allows the cow (and calf) to return from the reward pens to the start box. Dotted lines indicate gates that were closed during testing, but which could be opened when the cow (or calf) was herded to and from the arena.

The arena used for training and testing was located in the same barn as the home pens of the contact pairs and control calves (Fig. 3) and was identical to the apparatus reported by ref.^40^, so it was already familiar to the cows. The arena consisted of a start box, two reward pens and two return walkways; all sides were made of tubular metal bars, except the pen side dividing the two reward pens. The reward pens were each connected to the start box through a push gate and to each other through an opening in the wall (the “window”, Fig. 5). The resistance on the push gates was controlled using hydraulics. Throughout the experiment, the corresponding weight for each level of pressure was controlled (Table 1, see Supplementary Material 1 for description). The gates were fitted with rubber tires to increase cow comfort. Furthermore, a switch released the pressure on the gate when it was pushed more than half-way open; excessive pressure on the cow’s sides could thus be prevented and instead put on her shoulders and the back of the neck.

### Training

Three cow-calf pairs were excluded from the experiment before training began (one no-contact calf from block 1 and one part-time calf from block 4 were euthanized due to disease, and one no-contact cow from block 2 was euthanized due to an injury unrelated to the experiment). The total number of cows enrolled was therefore 81. Cow parity and calf sex were not randomly distributed across treatments, but the final distributions were similar across treatments (Table 3).

Approximately 2.5 weeks prior to the current study, cows had been trained to walk through the push gates for a companion study measuring their maternal motivation^40^. They were therefore familiar with the arena and the experience of walking through the push gates. Furthermore, their last experience with these push gates was at their maximum price, i.e., experiencing the heaviest weight on the push gate. Thus, the aim of training in the current study was to teach the cows that they now worked for either full contact to their calf (nursing possible) or only partial contact (limited calf contact through the “window”, nursing not possible), and that the weight on the gates was reset to the starting point.

Training took place Monday and Tuesday of week 8 (59.0 ± 6.2 d post-partum), in the morning before cow-calf pairs with reduced contact were reunited at 11:00 h. Treatment order in training and testing was balanced across blocks. Contact cows were deprived of calf contact for 1 h before training began by moving both reduced and unchanged contact calves to holding pens away from the home pens (Fig. 3). Before each session, the cow’s calf was fetched from the holding pen and herded to the arena where the calf was tethered to the end opposite to the push gate in one of the reward pens (Fig. 5, the same reward pen where the cow would have been rewarded in the previous study, ref.^40^). The other pen remained empty. The reward pen holding the calf remained consistent for each cow throughout training and testing but was balanced across cows.

The training process is visually represented in Supplementary Material 2. The first day of training consisted of a forced choice. Both push gates were closed with no added resistance. Balanced across treatment, cows were trained to access either full or partial calf contact first. The push gate leading to the other option was blocked by a board. The cow was fetched from the home pen and lead into the start box where she had 2 min to push through the available gate. If she did not pass the gate on her own accord, she was encouraged through by gently touching/clapping her body. Irrespective of how she passed the gate, she then received 2 min of undisturbed reward time, with either full or partial calf contact. If she was entering the empty reward pen, the calf would remain tethered, and it was noted whether she discovered the window and acknowledged her calf on the other side (Fig. 2). If she entered the reward pen holding the calf, the calf was released from its tether using a quick release allowing for free contact between cow and calf (including nursing if desired). Following the 2 min in the reward pen, the cow was herded back to the start box through the return walkway, and the calf was re-tethered if it had been released. If the cow had needed encouragement to pass the push gate on the first try, she got a second chance on the same gate. If she walked through on her own accord, or if she had been through the first gate twice, she got the option to pass through the opposite gate, while the first was blocked. If she had passed through the first gate without encouragement, but not the second gate, she got a second chance here; she could maximally pass through the gates three times in total. If the cow passed through one of the gates on her own accord, she proceeded to the next training step the following day. If not, this training step was repeated.

The second training day also consisted of a forced choice. The set-up was identical to the first training day, except that the pressure on the push gates was increased to 0.5 bar, the order of gate availability was switched compared to the first training day, and the cow now received 7 min of reward time in each reward pen. Additionally, the cow did not get any second chances on any of the gates. Cows met the pre-defined learning criterion when they passed through either of the gates on their own accord and proceeded to testing the following day. If not, this training step was repeated once more but only if the cow passed her first training step on her first day. The training period did not exceed three days.

### Testing: maximum price paid method (MPP)

Only cows meeting the learning criterion for training were included for testing (n = 68 cows). These cows were similarly distributed over treatments: 13 full time unchanged contact, 14 full-time reduced contact, 12 part-time unchanged contact, 15 part-time reduced contact, and 13 no-contact cows. Testing took place from the day after the cow had passed training (at the earliest Wednesday of week 8) until at the latest Friday of week 9. The cows were not tested during the weekend of week 8, and the testing period therefore consisted of a maximum of 8 days. Due to the experimental set-up, it was not possible to blind the cow handlers to contact treatment during the testing.

Similar to the training procedure, contact cows were completely deprived of calf contact for 1 h before testing by moving calves into the holding pens. After the deprivation period, the calf was herded to the testing arena and tethered in the assigned reward pen. Both push gates were closed; the pressure on the gate leading to the calf was set to 1.0 bar, while the other gate leading to the empty pen was set to 0.5 bar. The cow was then herded to the arena and had 2 min to walk through a gate from the moment she stepped into the start box.

If the cow entered the reward pen holding her calf, the calf was released from its tether using a quick release, and cow and calf were left undisturbed for 10 min. This reward time was selected to allow for a full nursing/suckling bout (7.8 to 10.1 min for dam-reared beef calves^23^; 6.4 to 8.8 min for dam-reared dairy calves^49^; 8.2 to 9.1 min for artificially-reared dairy calves^50^). Cow and calf were observed throughout the reward period; in case cows acted aggressively towards the calves, we would intervene. Fortunately, this was only necessary in very few (< 5) instances. In all those instances, a short loud shout by the handler directed towards the cow was enough to prevent further aggression. After the 10 min, unchanged contact pairs were led back to their home pen together, while reduced contact pairs were kept separated (cow in home pen, calf in holding pen) until their scheduled reunion at 11:00 h. No-contact cows returned to their free-stall pen in the other barn and their calves returned to their calf pen. Following every successful passing of the gate leading to full contact, the pressure was increased by 1.0 bar the following day, while the gate leading to the empty pen remained at 0.5 bar throughout testing.

If the cow entered the empty reward pen, the calf remained tethered, and the cow had undisturbed contact with the calf through the window for 10 min. Afterwards, the cow was herded back to her home pen. Unchanged contact calves remained in the reward pen for an additional 5 min, before they were returned to the home pen to avoid rewarding cows for choosing any other options than full contact. Reduced contact calves were herded directly to the holding pen.

If the cow did not pass a gate within 2 min, she was returned to her home pen. Again, unchanged contact calves remained in the reward pen for an additional 5 min before they were returned to the home pen, while reduced contact calves were returned to the holding pen.

For each pressure level, the cow had two chances to pass through the gate to full contact. If she failed her first attempt at a given weight, she received the same weight the following day. If she failed to pass again, it was interpreted that she had reached her maximum price, and she was not tested further. No cows were tested at a higher pressure point than 8.0 bar due to time constraints; cows and calves were permanently separated the following week.

For each testing session, the cow’s choice was recorded, as was the latency to make the choice, and time spent nursing the calf (if performed). Additionally, if the cow failed to reach full contact, it was recorded whether she first attempted to pass the gate to full contact before she chose one of the alternatives (partial contact or remaining in the start box).

### Statistical analyses

All statistical analyses were carried out in R (version 4.2.2^51^). *P*-values < 0.05 were considered significant. The effect of treatment on whether cows passed the learning criterion or not was analyzed using pairwise Fisher’s exact tests. Maximum price paid was analyzed using a Cox’s proportional hazards mixed effect model and the R packages coxme^52^ and survival^53^. This analysis was chosen as it allows for other factors than just the treatment groups to be included in the model. Furthermore, the analysis is based on the assumption, that survival probability is independent of time^54^. Using the Surv-function^53^, time was set to the highest pressure level successfully passed. Cows passing through 8.0 bar were censored to 0, as the test did not run for long enough to ensure that this was their actual maximum price. Cows not passing through 8.0 bar were censored to 1, as they reached their maximum price within the testing set-up. Survival probability was modelled as a function of treatment (full-time UC, full-time RC, part-time UC, part-time RC, or no contact), cow parity (primiparous or multiparous), calf sex (heifer or bull), calf age at first training day, position of the calf (left or right), and the nested random effects of block and pen group. Model fit was confirmed by assessing the significance of the integrated log-link test and by testing the proportional hazards assumption (using the cox.zph function^53^). To plot the survival curves, the model was also fitted using the coxph function^53^ with block and pen group combined and set as cluster.

As very few unchanged contact cows nursed their calves in the test, this variable was analyzed as a binary outcome using a Χ^2^ test. Whether cows utilized the option of partial contact rather than remaining in the start box was analyzed using binomial tests. For this analysis, only cows that had passed 1 bar of pressure and who had two failed attempts in a row were included (n = 23). Lastly, Fisher’s exact tests were used to analyze whether there was a relationship between cows attempting to pass the gate leading to full contact and their alternative choice (partial or no contact).

The latency for cows to make their choice was only descriptively analyzed, as only few cows from the no-contact and unchanged contact treatments made it to the highest resistance levels, preventing a good model fit.

Analyses were mainly carried out by the first author, who also handled the cows during testing. Data analyses were therefore not carried out blindly.

### Supplementary Information

Supplementary Information 1.

Supplementary Information 2.

## Data Availability

Datasets used in the current study are available from the Mendeley data repository: 10.17632/8fzkfn488p.2.
