# Supplementary material for: Dairy cows’ motivation to nurse their calves
Source: Sci Rep. 2024 Jun 14;14:13728. doi: 10.1038/s41598-024-64038-z (PMC11178926; doi:10.1038/s41598-024-64038-z)
Supplement: Supplementary file 1 — Supplementary Information 1. [file 41598_2024_64038_MOESM1_ESM.docx]

**Dairy cows’ motivation to nurse their calves**

**Supplementary material 1**

**Measuring weight on push gates**

To ensure that cows experienced the same weights irrespective of which side they had to walk through and which block they were in, the weight of the gates at each pressure level was measured throughout the experiment.

This was done using a custom-build wooden wedge, which was designed to push through the gates similarly to a cow (Figure 1). The wedge was connected through a rope to a load cell (“Bofors KRG-4”, 200 kg, Nobel Elektronik, Karlskoga, Sweden) (Figure 2) and the other side of the load cell was connected to a mechanical pulley system (Figure 3). The load cell was connected analogy to a weighing instrument (“BKI-5”, BLH Nobel, Karlskoga, Sweden), which measured the weight of the pull through the weighing cell. The load cell and weighing instrument were calibrated according to their protocol before the measurements were initiated.

The pressure on the gates was set to 1.0 bar, and the wedge was then pulled through the gate using the mechanical pulley. The highest weight recorded on the weighing instrument was noted down, and the process was then repeated three more times, for a total of four weight measurements. If these measurements differed more than 5 kg, the pressure on the gate was checked, and the measurements were redone. After the four measurements, the pressure was always controlled, to ensure that it had stayed consistent under the measurements. This process was repeated at all pressure levels up to 10.0 bar. The weights registered at each pressure level were then averaged and the standard deviations were found.

**FIGURES**


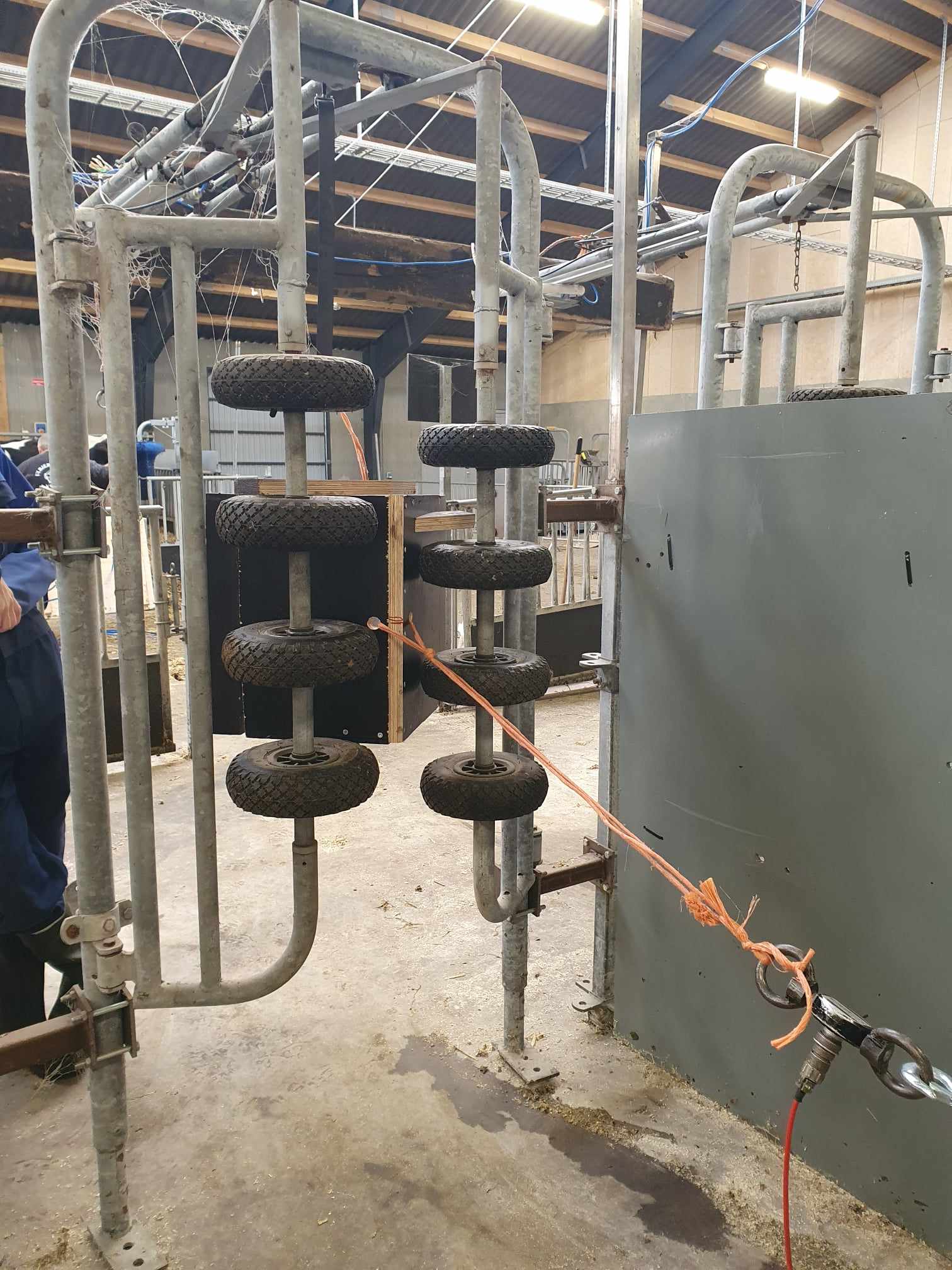


*Supplementary material 1, Figure 1: Custom-build wooden wedge to simulate cow walking through push gates. The wedge was fitted with a loose-fitting elastic rope to the top of the gate, to prevent it from falling down when the gate had been opened. In front, the wedge was connected to the load cell (through the orange rope) and from there to a mechanical pulley system.*

*
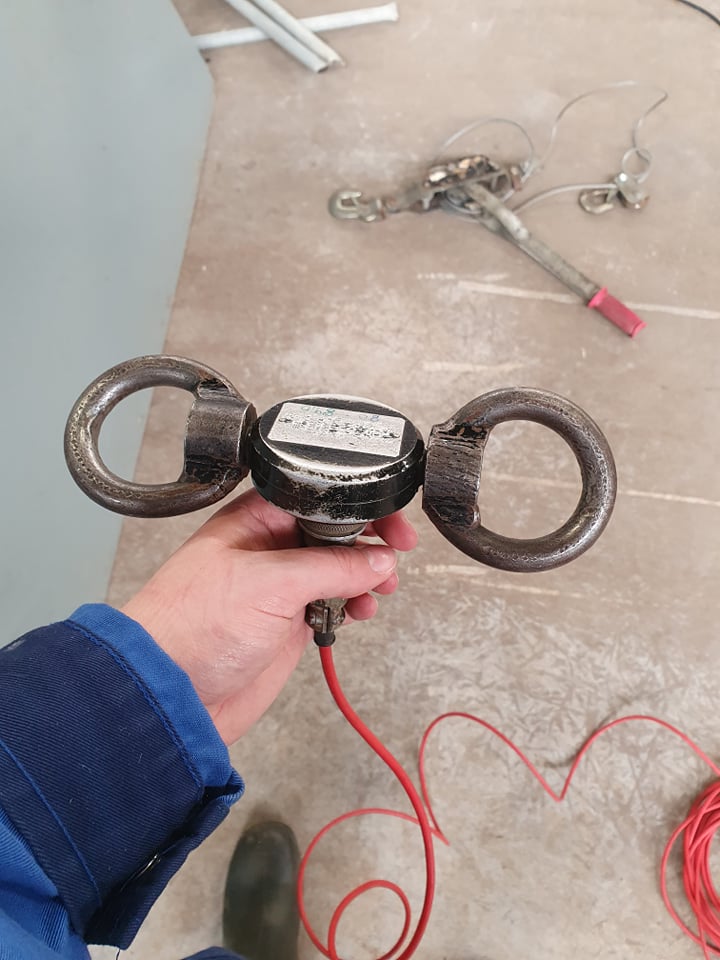
*

*Supplementary material 1, Figure 2: The load cell. The rope from the wedge was connected to one of the loops, while the steel wire from the mechanical pulley system was connected to the other loop.*

*
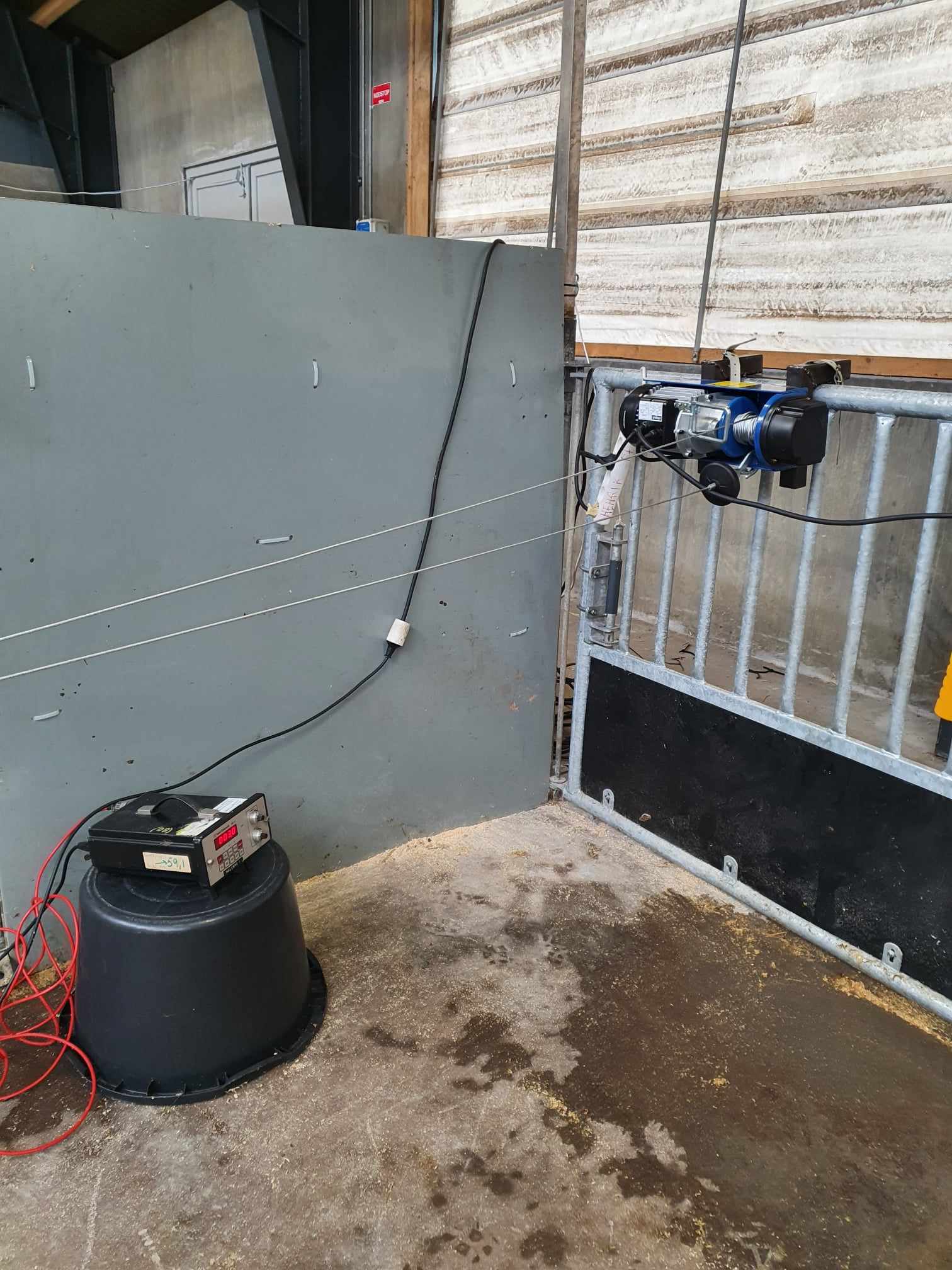
*

*Supplementary material 1, Figure 3: The weighing instrument (on the black bucket) and mechanical pulley system (on the back fence). The weighing instrument is connected to the load cell via the red cord. The steel wires from the pulley system are fastened to one end of the load cell.*
