# Supplementary material for: Dairy cows’ motivation to nurse their calves
Source: Sci Rep. 2024 Jun 14;14:13728. doi: 10.1038/s41598-024-64038-z (PMC11178926; doi:10.1038/s41598-024-64038-z)
Supplement: Supplementary file 2 — Supplementary Information 2. [file 41598_2024_64038_MOESM2_ESM.docx]

**Dairy cows’ motivation to nurse their calves**

**Supplementary material 2**

**
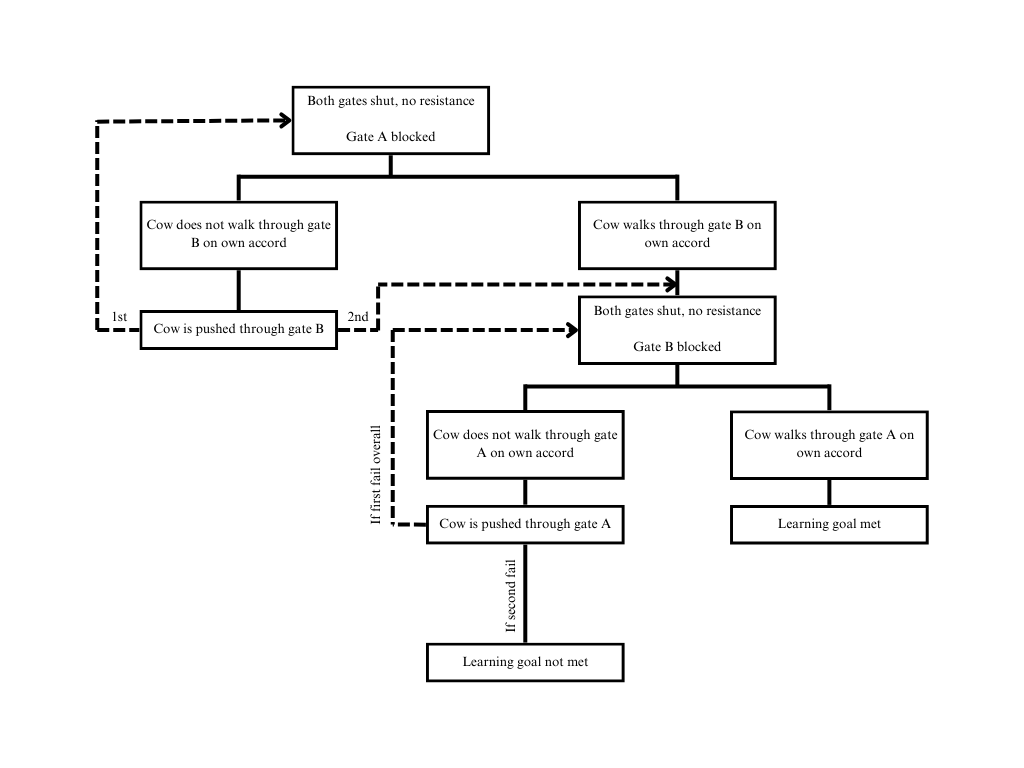
**

*Supplementary material 2, Figure 1: Flow chart depicting the training process on the first training day. Cows had previously been trained to pass through the push gates. One gate, either A or B, led to full calf contact, while the other led to partial calf contact. Both gates remained without resistance throughout the first training day. In a forced choice, the cow had to pass both gates. She had three chances in total to pass both gates. If she passed one of them on her own initiative, she had met the learning goal and continued to the next training step the following day. If not, this training day was repeated.*

**
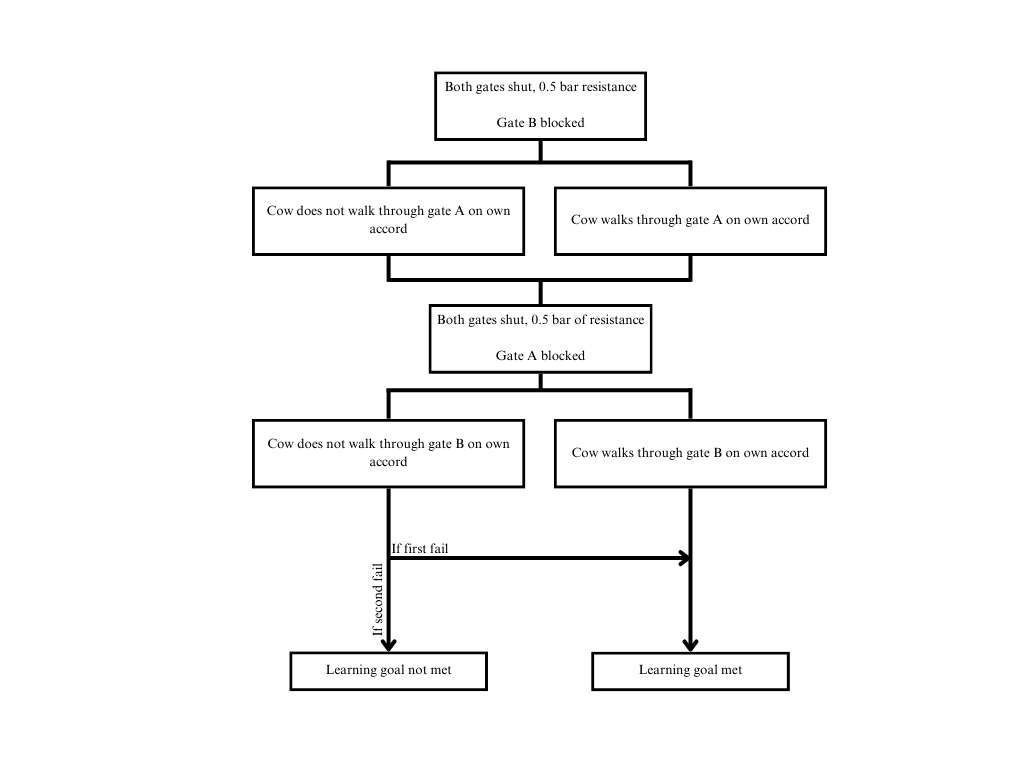
**

*Supplementary material 2, Figure 2: Flow chart depicting the training process on the second training day. One gate, either A or B, led to full calf contact, while the other led to partial calf contact. Which gate led to what remained consistent from the first training day. Both gates remained at 0.5 bar resistance throughout the second training day. In a forced choice, the cow had to pass both gates. She only got one chance per gate. If she passed one of them on her own initiative, she had met the learning goal and continued to the testing phase the following day. If not, this training day was repeated; however, only if the cow had passed the first training day on her first try. The training phase did not exceed three days total.*
